# Special Issue “Heterocyclic Compounds: Synthesis, Design, and Biological Activity”

**DOI:** 10.3390/ijms27125398

**Published:** 2026-06-15

**Authors:** Josef Jampilek

**Affiliations:** 1 Department of Chemical Biology, Faculty of Science, Palacky University Olomouc, Slechtitelu 27, 779 00 Olomouc, Czech Republic; josef.jampilek@gmail.com; 2 Institute of Chemistry, University of Silesia, Szkolna 9, 40-007 Katowice, Poland

Although basic organic chemistry defines organic compounds primarily as derivatives of the carbon–hydrogen backbone [1], it is the incorporation of heteroatoms that transforms this structural basis from an inert carrier into a highly specific entity [2,3,4]. The graphs in Figure 1 show the progress in publishing documents on heterocycles during the first quarter century of the new millennium. The hydrocarbon chain serves as a rigid or flexible scaffold, but the introduction of heteroatoms—whether classical (nitrogen, oxygen, sulfur) or rarer (phosphorus, selenium, boron)—and subsequent further substitution/replacement of carbon/hydrogen atoms, for example, with electronegative polar halogenated substituents, endows the molecule with a unique electronic profile and spatial arrangement [2,5,6,7,8,9,10,11].

The process of the isosteric or bioisosteric substitution/replacement of hydrocarbon fragments with heteroatoms represents a sophisticated strategy for fine-tuning pharmacodynamic and pharmacokinetic parameters. This approach, which can be applied both to linear aliphatic chains and—with even more pronounced effect—to cyclic systems, allows for the modification of the ionizability (p*K*_a_) of the molecule, its lipophilicity (log *P*) and the ability to form hydrogen bonds and π–π interactions, which are key factors for the bioavailability of molecules and interactions with the active sites of enzymes or receptors [8,12,13,14,15,16,17,18,19].

In modern molecular architecture, heterocyclic systems represent the so-called privileged scaffolds [20,21]. The static data is inexorable in this regard: more than 85% of physiologically active molecules are heterocycles or contain a single nitrogen atom in their complex structures [22], with approximately 60% of small anticancer chemotherapeutics containing a nitrogen-based heterocycle [23]. This phenomenon is not accidental; heterocycles provide an ideal balance between the rigidity necessary for molecular recognition and the chemical diversity that allows interactions with a wide range of biological targets [24,25,26,27,28,29,30,31,32]. In addition to the modification of the hydrocarbon backbone with heteroatoms, the subsequent substitution of the skeleton is also important. In this context, the replacement of hydrogen with fluorine atoms stands out, which has become one of the most important and most frequently used strategies in modern medicinal chemistry and agrochemistry. Fluorine is not just an “additional atom”; its introduction into a molecule can dramatically change its biological, physicochemical, and pharmacokinetic properties [33,34,35,36,37,38,39,40,41]. The main reasons for the popularity of this “magic atom” lie in (i) bioisosterism (the van der Waals radius of fluorine is very close to that of hydrogen, but dramatically changes the electronic properties of the surroundings); (ii) metabolic stability (the introduction of fluorine into a position oxidized by enzymes (e.g., cytochromes P450) can block the metabolism of the drug and prolong its half-life); and (iii) modulation and lipophilicity (fluorine is the most electronegative element—its introduction removes electron density from the heterocyclic ring, which reduces the basicity of nitrogenous bases, which affects solubility and membrane permeability) [34,38,40,42,43,44,45,46,47,48,49,50].

Although a wide range of synthetic methodologies is available—from the now classic metal-catalyzed cross-coupling reactions to advanced cyclization cascades—current trends in research on heterocycle construction are directed toward the search for extremely efficient, sustainable (“green”), and selective methods for the preparation and modification of these structures, such as (i) the direct activation and functionalization of C–H bonds; (ii) photoredox and electrochemical catalysis; and (iii) flow chemistry and automation [51,52,53,54,55,56,57,58,59,60,61,62,63,64,65,66,67,68]. In addition to the above synthetic approaches, it is necessary to mention the molecular editing of heterocycles as a unique and innovative transformation chemical strategy that economically replaces the time-consuming, multi-step total synthesis of heterocycles while enabling structural diversification [69,70,71,72].

Despite these advances and successes in synthesis, nature remains an unsurpassed source of structural complexity. Many heterocyclic compounds and pharmacophores have been isolated from natural sources, where evolution over millions of years has optimized their structure for specific biological functions [13,73,74,75,76]. Modern medicinal chemistry embraces this insight and uses the principles of biomimetic design to create new, more potent compounds, whether for human medicine or for precision agrochemical applications that require high potency and selectivity without environmental burden [77,78,79,80,81].

The type, quantity, and, above all, topology of heteroatoms in a molecule determine not only its physicochemical properties, but also define its “binding signature”. Knowledge of the thermodynamics and kinetics of interactions between these molecules and their biological environment is the driving force of contemporary biological sciences, including chemical biology. In the era of personalized medicine and precision agriculture, understanding how substitution effects on the heterocyclic nucleus influence ADME (absorption, distribution, metabolism, and excretion) profiles is an absolute necessity for further scientific and technological progress [9,82,83,84].

It can be said that molecular interactions between biological target and therapeutic agent are primarily caused by heteroatoms, which are the key to understanding biological interactions and are the architects of biological activity; therefore, heterocycles do not only form the basis of drugs, they are features that profile druggability [85,86] so that the molecule shows a high druglike score [87,88,89].

This Special Issue is dedicated to the topics of the synthesis, isolation, structural aspects, and analysis of heterocycles and the design, discovery, and development of biologically active heterocyclic compounds and their interactions, including their eco(toxicological) effects. This issue includes 10 articles, three reviews, and seven original scientific papers. Pintea et al. (contribution 1) publish an extensive review of pyrazolo[5,1-*c*][1,2,4]triazoles as bioactive compounds and their diverse applications in pharmaceuticals, agrochemicals, dyes, polymers, cosmetics, etc. Similarly, Asproni et al. (contribution 2) investigate tricyclic molecules based on pyridazin-3(2*H*)ones in terms of their pharmacological properties, with an emphasis on anti-inflammatory, analgesic, antitumor, antimicrobial, antiviral, cardiovascular protective, and antiulcer activities. Bioanalysis is increasingly gaining prominence due to the need to assess drug concentrations in the body; the bioanalysis of body fluids plays a key role in clinical diagnostics, pharmaceutical research, forensic science, and biomarker discovery. In a review article, Masár et al. (contribution 3) discuss bioanalysis using capillary electrophoresis and its modifications, which have proven to be efficient, affordable, and miniaturized alternatives for the analysis of small organic and inorganic molecules in urine, blood, saliva, and cerebrospinal fluid. Among the original scientific (experimental) papers, one can find synthetic works, where some biological activities (low-calorie sweetener (contribution 4), anti-inflammatory (contribution 5), antibacterial (contribution 6) or photodynamic effects (contribution 7)) of compounds obtained using innovative synthetic approaches were additionally tested, but also explicitly pharmaceutical-chemical works with targeted molecules either as antitumor drugs (protein kinase inhibitors) (contribution 8), Fe(II) tris(1-pyrazolyl)methane complexes for the treatment of hereditary psychic disorders (contribution 9), or type II flavin-based photosensitizers (contribution 10).

## Figures and Tables

**Figure 1:**
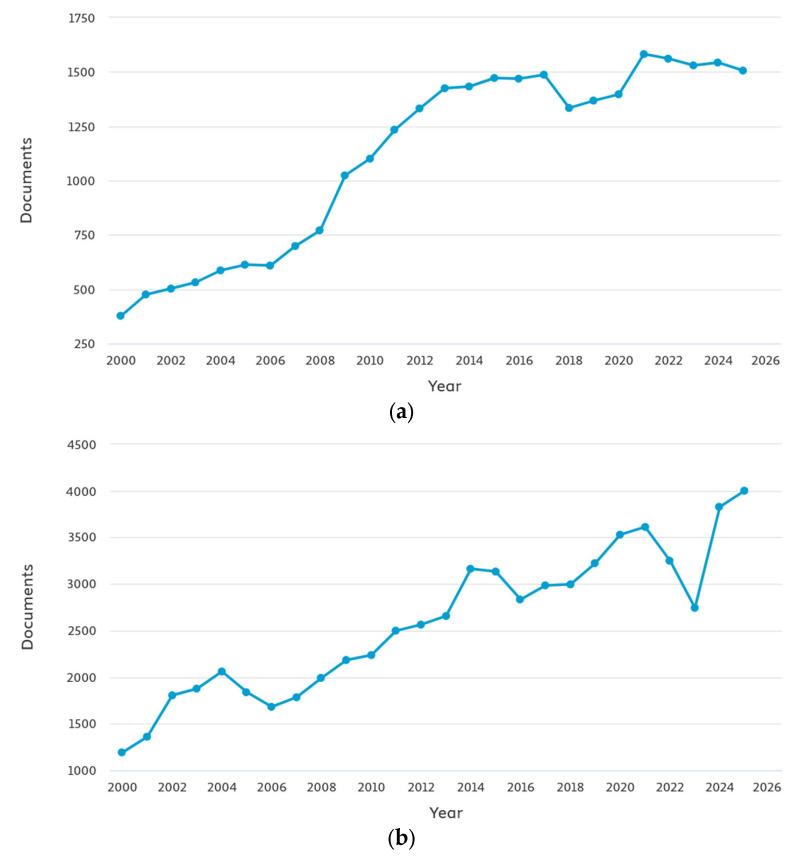
Graphs documenting the number of published documents in the years 2000–2025 in the Scopus database as of 1 June 2026: (**a**) keyword “heterocycle”; (**b**) keyword “heterocyclic”.
